# Age is associated with increased mortality in the RETTS-A triage scale

**DOI:** 10.1186/s12877-019-1157-4

**Published:** 2019-05-23

**Authors:** T. Ruge, G. Malmer, C. Wachtler, U. Ekelund, E. Westerlund, P. Svensson, A. C. Carlsson

**Affiliations:** 1 Department of Emergency Medicine, Huddinge, Karolinska University Hospital, Stockholm, Sweden; 2 Department of Medicine, Solna, Karolinska Institutet, Stockholm, Sweden; 3 Division for Family Medicine and Primary Care, Department of Neurobiology, Care Sciences and Society, Karolinska Institutet, Stockholm, Sweden; 4 Faculty of Medicine, Department of Clinical Sciences Lund, Emergency Medicine, Lund University, Lund, Sweden; 5 Department of Clinical Sciences, Danderyd Hospital, Karolinska Institutet, Stockholm, Sweden

**Keywords:** Triage, RETTS-A, Mortality, Age, Emergency department, Rapid emergency triage and treatment system– adult, Cohort study

## Abstract

**Background:**

Triage is widely used in the emergency department (ED) in order to identify the patient’s level of urgency and often based on the patient’s chief complaint and vital signs. Age has been shown to be independently associated with short term mortality following an ED visit. However, the most commonly used ED triage tools do not include age as an independent core variable. The aim of this study was to investigate the relationship between age and 7- and 30-day mortality across the triage priority level groups according to Rapid Emergency Triage and Treatment System – Adult (RETTS-A), the most widely used triage tool in Sweden.

**Methods:**

In this cohort, we included all adult patients visiting the ED at the Karolinska University Hospital, Sweden, from 1/1/2010 to 1/1/2015, *n* = 639,387. All patients were triaged according to the RETTS-A and subsequently separated into three age strata: 18–59, 60–79 and ≥ 80 years. Descriptive analyses and logistic regression was used. The primary outcome measures were 7- and 30-day mortality.

**Results:**

We observed that age was associated with both 7 and 30-day mortality in each triage priority level group. Mortality was higher in older patients across all triage priority levels but the association with age was stronger in the lowest triage group (*p*-value for interaction = < 0.001). Comparing patients ≥80 years with patients 18–59 years, older patients had a 16 and 7 fold higher risk for 7 day mortality in the lowest and highest triage priority groups, respectively. The corresponding numbers for 30-d mortality were a 21- and 8-foldincreased risk, respectively.

**Conclusion:**

Compared to younger patients, patients above 60 years have an increased short term mortality across the RETTS-A triage priority level groups and this was most pronounced in the lowest triage level. The reason for our findings are unclear and data suggest a validation of RETTS-A in aged patients.

## Background

Triage, initially developed in military medicine as a way to quickly allocate appropriate medical resources, is now used internationally in many clinical settings especially in the emergency department (ED) [1]. As EDs face increasing number of patient visits and crowding, triage has been an important tool to identify and prioritize patients with need of acute treatment and to reduce waiting time at the ED [2]. Initial triage priority is determined by assessment of patients’ vital signs, followed by algorithm-based assessment of symptoms and signs. Deviations in vital signs are associated with increased mortality and mortality increases with high triage priority level [2, 3].

Elderly patients are a particularly vulnerable population in the EDs and may not be adequately served by current triage tools. The population of already overcrowded EDs is aging in many western countries. Elderly patients have multiple health problems, and they are more frequently admitted and experience adverse outcomes after they are discharged from the ED [4–6]. There is evidence suggesting that elderly patients have a higher risk of being under-triaged, have longer boarding times and that serious medical conditions may go unrecognized [5, 7, 8]. Specifically, under-triage of elderly trauma patients may result in adverse outcome for critically ill patients [9].

In most triage systems, age is not included as an independent core variable for assessing clinical urgency. Importantly however, it has been shown that increased age is significantly and independently associated with 1 day mortality in ED patients [3]. The aim of this study was therefore to investigate the relationship between age and 7- and 30-day mortality in separate triage priority level groups. Patients included in this study were all triaged according to RETTS-A (https://predicare.se/), the most common used triage system in Sweden.

## Methods

### Study design and setting

This study was a cohort study including data from adult patient visits to two large University hospitals EDs in Stockholm, Sweden. Patients were triaged according to the five level RETTS-A triage tool as part of day-to-day operation. Upon arrival at the ED, the patient was registered at the front desk for either further triage or not.

In the subsequent triage process, patient cause of contact and vital signs as well as medical history were collected. RETTS-A triage includes 43 predefined Emergency Symptoms and Signs (ESS) and the patients cause of contact was coordinated with the ESS protocol. Final triage priority was given by a nurse as a result of a careful and combined evaluation of the ESS protocol as well as the vital signs of the patients where the most medical acute of these decided patient final triage priority. The triage priorities are in order of acuity: Red, orange, yellow, green and blue, with red being the highest and blue being the lowest priority. Blue priority is only assigned to the patients who are not assessed to be in need of any further emergency care.

### Study population

Inclusion critera were all adult patients visiting the ED at the Karolinska University Hospital, Sweden, from 1/1/2010 to 31/12/2016, *n* = 639,387. Exclusion criteria were:

1. No full documentation of all variables (missing data),
2. Patients deceased upon arrival to the ED
3. Patients whose first triage priority was blue
4. Patients with a length of ED length of stay (ED-LOS) > 4000 min

### Data collection

Data on the independent variables were collected by extraction from central hospital systems. Age and sex can be read directly from the ID number required for health service by every Swedish resident. Information on chief complaints, and triage priority was obtained from patient record systems. Total ED-LOS, if the patient was given prehospital care or not and if the patients were admitted to hospital or not was obtained by extracting this information from the hospital management system. Since all these sources of information are extensively used as part of day-to-day operations at the hospital, the data is validated and checked routinely as a consequence of hospital operation and medical assessment. We have also made graphical representations of data to identify potential systematic patterns and outliers. Information on patient survival as dependent variable was extracted from the Swedish population register, administrated by the Swedish Tax Agency, which includes every Swedish resident and has a high validity and completeness. Thus there was a near complete follow-up of 7- and 30 day mortality for every patient visiting the EDs included in this study.

### Outcomes

Primary outcomes were 7- and 30-day mortality, counted from registration to the ED.

### Statistical analyses

Baseline characteristics includes descriptive analyses presented with means and standard deviations. Patients were categorized in three different age strata: 18–59 years, 60–79 years and ≥ 80 years. Pearson Chi-square test and ANOVA was used for comparison across groups. Multiple logistic regression models were performed to investigate multiplicative interactions with age and the relationship between age and mortality for each RETTS-A triage priority level, resulting in odds ratios (OR) with 95% confidence intervals (CI). The crude model (Model A) included age, sex and the outcome, Model B included Model A and ED-LOS, the ten most common chief complaints, and prehospital care and finally Model C included Model B and in-hospital care. Two-sided *p*-values <  0.05 were considered significant. Statistical analyses were carried out in STATA version 13.

## Results

### Baseline characteristics

Baseline characteristics of the study populations are presented in Table 1. Compared to younger patients, patients ≥80 years were more often admitted to hospital care (*p* <  0.001), had more often a high triage priority (*p* <  0.001) and had longer ED-LOS (*p* <  0.001). Mortality, including 7- and 30-days mortality, was higher among elderly patients compared to younger patients (*p* < .001) and increased with triage priority level (*p* <  0.001).

**Table 1:** Baseline characteristics

Characteristic	Age 18–59 years	Age 60–79 years	Age ≥ 80 years	*p*-value
Number of patients (% of total *n* = 639,387)	58	28	14	
Age (years, median ± STDEV)	39 ± 12	69 ± 5	87 ± 5	
Gender (% per age strata, women)	56	49	60	< 0.001
Prehospital care (% per age strata)	58	55	52	< 0.001
ED- Length of stay (minutes, median, IQR)	202, 168	231, 184	263, 200	< 0.001
Admitted to hospital (%, per age strata)	19	41	51	< 0.001
Triage (%, per age stratum)				
Red	4	7	9	< 0.001
Orange	12	19	23	< 0.001
Yellow	38	45	46	< 0.001
Green	46	29	22	< 0.001
Outcomes				
7-day mortality (%, per age strata)	0.1	1	3	< 0.001
30-day mortality (%, per age strata)	0.3	3	7	< 0.001
7-day mortality (%, per age strata, Red/Green)	1.3/0.01	5.77/0.2	11.7/0.6	< 0.001
30-day mortality (%, per age strata, Red/Green)	2.2/0.06	10.5/0.90	21.2/2.9	< 0.001

### Primary results

The results of the multiple logistic regression models for 7 and 30 day mortality are presented in Table 2 and Table 3, respectively. Both 7 and 30-day mortality OR increased with increased age in each of the triage priority levels. Age associated mortality OR was consistently higher for lower triage priorities than for higher priorities, both for 7 and 30 day mortality. The strongest association between older age and mortality was observed in the lowest (green) triage priority level. The pattern of increased mortality among older patients, with larger ORs in lower priority patients, was observed in all models also after correcting for all included potential moderators (gender, LOS, cause of contact, prehospital care and in-hospital care). There was a multiplicative significant interaction between age group and triage level (data not shown in tables).

**Table 2:** 7-day mortality per triage group, logistic regression analysis results. Model A (age, gender), model B (Model A and LOS, cause of contact, and prehospital care), model C (Model B and in-hospital care). Odds Ratios (OR) compared to age group 18–59 years. ***: *p* < 0.001

Variable	Model A Mortality 7d OR (95% CI)	Model B Mortality 7d OR (95% CI)	Model C Mortality 7d OR (95% CI)
Triage group: Red			
Age			
60–79 years	4.7 (4.0–5.5) ***	4.0 (3.4–4.7) ***	3.5 (2.9–4.1) ***
≥ 80 years	10.2 (8.7–11.9) ***	7.9 (6.7–9.3) ***	6.8 (5.7–8.0) ***
Triage group: Orange			
Age			
60–79 years	5.8 (4.7–7.2) ***	4.1 (3.3–5.2) ***	3.5 (2.8–4.3) ***
≥ 80 years	15.4 (12.5–19.0) ***	7.9 (6.3–9.8) ***	6.6 (5.3–8.2) ***
Triage group: Yellow			
Age			
60–79 years	7.6 (6.2–9.4) ***	5.6 (4.5–6.9) ***	4.5 (3.6–5.6) ***
≥ 80 years	19.6 (15.9–24.1) ***	9.4 (7.6–11.7) ***	7.4 (5.9–9.1) ***
Triage group:Green			
Age			
60–79 years	15.4 (9.4–25.3) ***	10.7 (6.5–17.7) ***	8.7 (5.2–14.4) ***
≥ 80 years	52.4 (32.3–85.1) ***	21.8 (13.1–36.2) ***	16.2 (9.7–27.1) ***

**Table 3:** 30-day mortality per triage group, logistic regression analysis results. Model A (gender, age), model B (Model A and LOS, cause of contact, and prehospital care), model C (Model B and in-hospital care) . Odds Ratios (OR) compared to age group 18–59 years. ***: *p* < 0.001

Variable	Model A Mortality 30d OR (95% CI)	Model B Mortality 30d OR (95% CI)	Model C Mortality 30d OR (95% CI)
Triage group: Red			
Age			
60–79 years	5.2 (4.6–5.9) ***	4.3 (3.8–4.9) ***	3.7 (3.2–4.2) ***
≥ 80 years	12.0 (10.6–13.6) ***	9.1 (8.0–10.4) ***	7.7 (6.8–10.4) ***
Triage group: Orange			
Age			
60–79 years	5.5 (4.9–6.2) ***	4.1 (3.6–7.6) ***	3.5 (3.1–3.9) ***
≥ 80 years	12.6 (11.2–14.2) ***	7.0 (6.1–7.9) ***	5.9 (5.2–6.7) ***
Triage group: Yellow			
Age			
60–79 years	7.8 (7.1–8.7) ***	6.1 (5.5–6.7) ***	5.0 (4.5–5.6) ***
≥ 80 years	17.6 (15.8–19.5) ***	9.8 (8.8–10.9) ***	7.9 (7.0–8.8) ***
Triage group:Green			
Age			
60–79 years	15.1 (12.1–18.7) ***	11.8 (9.5–14.7) ***	9.7 (7.7–12.1) ***
≥ 80 years	50.6 (40.9–62.6) ***	27.0 (21.6–33.8) ***	20.6 (16.5–25.9) ***

## Discussion

### Main findings

Older age was associated with mortality, across all the triage priority level groups according to RETTS-A. The strongest association with mortality was observed in the lowest (green) triage priority group.

### Comparison with previous studies

The most common triage systems are based on a five-level structured scoring system, they have been proven valid and reliable methods for assessment of the severity of incoming patients’ conditions [10] and the link between high triage priority level (high level of clinical urgency) and increased mortality, high probability for admission for in-hospital stay as well as length of stay at the hospital are well documented [2]. Age is not included as a core variable in most common triage systems including RETTS-A. Among the most common used triage systems only the Emergency Severity Index (ESI) has been validated in patients > 65 years. Most others have not been tested [5] and neither has triage priority related mortality been adjusted for age or gender [2]. However age has been shown to be significantly and independently associated with 1 day mortality in ED patients [3]. In addition, increasing age has previously been shown to be an independent predictor of mortality following trauma [11] and furthermore, age has been associated with increased mortality also in studies of a delimited condition including patients suffering from acute coronary syndromes [12] and stroke [13]. Age was also associated also with increased in-hospital mortality in nonsurgical admitted patients [14] and in 5583 unsorted patients admitted to hospital care [15].

### Possible mechanisms

The increased mortality rates observed in the aged patients visiting the ED in our study were of similar magnitudes as shown before [6, 16]. In the here presented data, patients in the oldest age group and with the lowest triage priority had a 16 and 21-fold increased risk for 7 and 30-days mortality compared to patients in the youngest age group. We have not been able to find results of similar levels in the literature.

In comparison with the youngest age group, the difference in the mortality risk for the other two age groups increased as triage priority level declined. Recently the performance of the Manchester Triage system was validated in older ED-patients. Here the authors observed a worse predictive ability of the triage tool for in-hospital mortality, particularly in medical and elderly patients [17]. Additionally, and in accordance with our findings, a higher mortality was observed in elderly patients triaged with low urgency compared to younger counterparts, 1.7 and 0.1% for patients triaged as green, respectively [17].

Emergency department triage tools, including RETTS-A, rely heavily on vital signs upon arrival. It is known that vital sign cut-offs are unreliable for elderly patients, which may affect the validity of RETTS-A and similar triage systems in elderly patients [9]. The risk for under triage of elderly patients is therefore high and can be one explanation behind the increased mortality observed in elderly patients. Another explanation could be the possible difficulty for ED-staff to identify medical urgency in elderly patients [6, 8]. For example, Rutschmann et al. observed an underlying acute medical illness in around 50% of low triaged elderly patients visiting the ED with non-specific complaints [18].

Frailty includes a combination of musculoskeletal, neuroendocrine, nutritional, and immunologic defects which results in a phenotype characterized by a loss of muscular strength and a decline in functional ability [19, 20]. Increased age is associated with increased patient frailty and compared to no frail patients, frail patients have increased in-hospital mortality, elevated incidence of comorbidity as well as comorbidity severity, increased length of hospital stay and worse long-term outcomes [21, 22]. Because elderly patients are more likely to be frail than younger patients, frailty may at least partially explain the relationship between increasing age and increased risk for mortality seen in our study. Further studies need to be done to identify if age is an overly simplistic measure to understand outcomes in geriatric patients in the ED, and if frailty might be a better predictor than age.

### Strengths and limitations

The major strength of this study was the large number of patients included in this and that we were able to link clinical data on the ten most common causes of contact, prehospital care, in-hospital care and triage priority from individual electronic emergency department patient records to mortality. We used a stringent study design that allowed us to specifically test the relationship between age and risk for mortality in each of the triage priority groups and, to our knowledge, this is the first study explore the association between age and mortality within RETTS-A triage levels.

Our study has some important limitations. Firstly, the main aim for RETTS triage is to identify medical urgency among ED patients and not to predict mortality. However, it is complicated to validate triage tools since there is no gold standard for the degree of medical urgency. Therefore surrogate markers such as rate of hospital admission and mortality rate are used to assess validity [23]. Secondly, we did not have access to all relevant underlying diagnoses and comorbidities of the included patients. However, underlying diagnosis and comorbidities are not used in RETTS-A. Another limitation is that this is an observational study. However, many clinical trials often exclude individuals with multiple morbidities. In the current study, we included all patients seeking care at the ED at two large hospitals in our analyses, which means that the findings are more representative of the variety of patients encountered in clinical practice at ED than investigation with strict inclusion and exclusion criteria.

In this study we chose under 60 years and over 80 years of age as clinically meaningful cut-off points. To our knowledge there is no established standards for what age is considered as a proper limit for considering a patient to be geriatric or elderly, in the literature different age cut offs have been suggested ranging from 45 to 80 years of age [24–30].

## Conclusion and implications

In our study we have identified that age is strongly associated with mortality in all triage priority level groups defined by RETTS-A and that this association is more substantial in the lower triage priority groups. Elderly patients, specifically those above 80 years of age, are at higher risk for mortality no matter what triage priority group that they qualify for. We believe that it is important to validate RETTS-A in elderly. Future research should investigate if the relationship between age and mortality risk holds even when other triage tools are used. Additionally, there is a clinical need for intervention studies to test if addressing age in the ED can improve patient outcomes.

## Abbreviations

AIS: abbreviated injury scale
CT: computed tomography
ED: emergency department
GCS: Glasgow coma scale
IBD: injury database
LOS: length of hospital stay
PAS: patient administrative system
RETTS-A: rapid emergency triage and treatment system
TBI: traumatic brain injury
TFSI: The Trauma Specific Frailty Index
